# *IGFBP3 *mRNA expression in benign and malignant breast tumors

**DOI:** 10.1186/bcr1634

**Published:** 2007-01-08

**Authors:** Zefang Ren, Aesun Shin, Qiuyin Cai, Xiao-Ou Shu, Yu-Tang Gao, Wei Zheng

**Affiliations:** 1 Department of Medicine, Vanderbilt Epidemiology Center, Vanderbilt-Ingram Cancer Center, Vanderbilt University, S-1121 MCN, 1161 21^st ^Ave. S, Nashville, TN 37232-2587, USA; 2 Department of Medical Statistics and Epidemiology, School of Public Health, Sun Yat-sen University, 74# Zhongshan Rd 2, Guangzhou 510080, People's Republic of China; 3 Department of Epidemiology, Shanghai Cancer Institute, 2200/25 Xie Tu Rd, Shanghai 200032, People's Republic of China

## Abstract

**Introduction:**

Most previous studies have focused on evaluating the association between circulating insulin-like growth factor binding protein 3 (IGFBP-3) levels and breast cancer risk. Emerging evidence over the past few years suggests that IGFBP-3 may act directly on mammary epithelial cells.

**Methods:**

To understand the role of IGFBP-3 in breast tumorigenesis, we investigated *IGFBP3 *mRNA expression levels in benign and malignant breast tumors and their adjacent normal tissues using real-time quantitative PCR.

**Results:**

Cancer tissues had significantly lower *IGFBP3 *expression than benign tumor tissues (*p *< 0.001). *IGFBP3 *expressions in both tumor and adjacent tissues were higher in patients who had proliferative benign tumors than in those who had non-proliferative benign tumors. Among patients with benign breast disease, *IGFBP3 *expression in the tumor was significantly higher than that in their adjacent normal tissue. There were no apparent associations of *IGFBP3 *expression in cancer tissues with either overall survival or disease-free survival in a cohort of 521 patients with breast cancer.

**Conclusion:**

Our findings suggest that the expression level of *IGFBP3 *in breast tissues may be involved in breast tumorigenesis.

## Introduction

The insulin-like growth factor (IGF) system is important in regulating cell proliferation, differentiation, apoptosis, and transformation [1]. *In vitro *studies have consistently shown that members of the IGF family not only regulate the growth of various cancer cells but also interact with other cancer-related molecules [1,2]. Insulin-like growth factor binding protein 3 (IGFBP-3) is the most abundant IGFBP in circulation [3]. IGFBPs can either suppress or enhance the action of IGFs. Because the affinity of IGFBPs for IGFs is the same as or greater than that of the IGF-I receptors, the presence of IGFBPs could inhibit IGF activity by decreasing levels of free IGFs available to activate the receptor [2,3]. However, the binding of IGFBPs to IGFs protects IGFs from proteolytic degradation, thus enhancing the action of IGFs by increasing their bioavailability in local tissues [3]. In meta-analyses of the association between the blood concentration of IGFBP-3 and breast cancer risk, high blood levels of IGFBP-3 were associated with an increased risk of premenopausal breast cancer but not with that of postmenopausal breast cancer [4,5]. However, a recent nested case-control study in the European Prospective Investigation into Cancer and Nutrition (EPIC) showed an increased risk for breast cancer with high circulating IGFBP-3 after 50 years of age [6]. No association was observed in younger women [6].

IGFBP-3 has been shown to induce apoptosis of breast cancer cells and inhibit breast cell growth in IGF-independent ways [3,7,8]. Many tissues express *IGFBP3 *[3], and breast tissue also expresses it [9-11]. Most previous studies have focused on evaluating circulating IGFBP-3, and almost none have systematically investigated the level of this important protein in breast tissues from patients with invasive cancer and benign breast diseases (BBDs). In the present study we evaluated the expression level of the *IGFBP3 *gene in breast tissues from patients with breast cancer or BBD, and investigated potential associations between *IGFBP3 *mRNA expression levels and the clinicopathologic features of the tumors and breast cancer survival.

## Materials and methods

### Study subjects and tissue samples

Included in this study was a subset of patients who participated in the Shanghai Breast Cancer Study [12]. These patients were diagnosed with breast cancer or BBD between 1996 and 1998. Study patients were recruited through a rapid case ascertainment system established through a network of major hospitals that treat more than 80% of breast cancer cases in urban Shanghai. A total of 1,602 women who were diagnosed with a primary breast cancer were identified. Of these, 1,459 (91.1%) participated in the Shanghai Breast Cancer Study. During the operation, tissue samples were obtained from the tumor and another section (if available) from the distal edge of the resection. These samples were snap-frozen in liquid nitrogen as soon as possible, typically within 10 minutes. Samples were stored at -70°C until the relevant assays were performed.

All patients were interviewed at the time of recruitment. A structured questionnaire was used in this study to collect information on demographic factors, menstrual and reproductive history, hormone use, previous disease history, family history of cancer, physical activity, tobacco and alcohol use, and usual dietary habits. All participants were measured for current weight, circumferences of the waist and hips, and sitting and standing heights. Medical charts were reviewed using a standard protocol to obtain information on cancer treatment, clinical stages and cancer characteristics, such as estrogen and progesterone receptor status. Two senior pathologists reviewed pathology slides to confirm the diagnosis for breast cancer or BBD. BBDs were classified on the basis of the published criteria developed by Page and colleagues [13].

All of the patients with breast cancer of the main study were followed until July 2005 by a combination of in-person or telephone contacts and record linkage to the death certificates kept by the Shanghai Vital Statistics Unit [14,15]. In all, 89.2% of patients successfully completed the follow-up interview either in person or by telephone. For those who could not be contacted in person or by telephone, linkage to the death certificate data was completed to obtain information on the date and cause of death. Subjects who had no match in the death registry were assumed to be alive on 30 December 2004, 6 months before the linkage, to allow for a possible delay of entry of the death certificates into the registry.

### Laboratory methods

Total RNA was extracted from tissue specimens by homogenization in TRIzol solution (Gibco BRL, Carlsbad, CA, USA), phase separation, precipitation, and washing in accordance with the manufacturer's instructions. The quality and quantity of RNA were measured by spectrophotometric analysis. RNA was reverse-transcribed in a final volume of 15 μl containing 0.15 μg RNA and 1 × RT-PCR buffer, 5.5 mM MgCl_2_, dNTPs (each at 500 μM), 2.5 μM random hexamers, 0.4 U/μl RNase inhibitor, and 3.125 U/μl MultiScribe reverse transcriptase (Applied Biosystems, Foster, CA, USA). The mixture was incubated at 25°C for 10 minutes, at 37°C for 120 minutes and 95°C for 5 minutes.

The primers and probes used for the *IGFBP3 *(Hs00426287) and *β-actin *(Hs99999903) genes were obtained from Applied Biosystems. Quantitative real-time PCR was performed with a 384-well optic plate on an ABI PRISM^® ^7900HT Sequence Detection System (Applied Biosystems). A total reaction volume of 5 μl containing 2.2 μl of cDNA template (1:10 dilution), 1 × TaqMan Universal PCR Master Mix (without uracil-N-glycosylase), and 1 × Gene Expression Assay Mix including the primers, and marked probes from Applied Biosystems Assays-on-Demand services. The thermal cycling conditions were as follows: 95°C for 10 minutes to activate AmpliTaq Gold enzyme, followed by 40 cycles of 95°C for 15 seconds and 60°C for 1 minute.

Every sample was tested in triplicate. Two control samples were used in each plate to monitor variation between plates, which was found to be smaller than 5% in our study. The threshold cycle (*Ct*) was determined as 0.1 on the basis of the amplification linear area of both the *IGFBP3 *and *β-actin *genes. The normalized quantity of *IGFBP3 *was calculated as 2^-Δ*Ct*^, where Δ*Ct *was obtained directly by subtracting *Ct *for the target gene from *Ct *for the *β-actin *gene. The final result was expressed as 2^-Δ*Ct *^× 1,000.

The initial samples (batch 1) included in the current analysis were tumor tissues from 142 patients with primary breast cancer and 96 patients with BBD. Also included in the study were paired adjacent normal tissues from 63 patients with cancer and 60 patients with BBD. For survival analysis, we analyzed tumor tissue samples from an additional 379 patients with breast cancer (batch 2) to enhance statistical power.

### Statistical analysis

The data were skewed to the high value; log-transformed *IGFBP3 *expression levels were used therefore for analyses. Linear regression models were used to compare differences in *IGFBP3 *expression between comparison groups with adjustment for age (continuous variable) and menopausal status. The paired *t *test was used for comparing *IGFBP3 *expression levels between tumor tissues and their adjacent normal tissues. To evaluate associations of *IGFBP3 *expression with breast cancer survival, patients were grouped on the basis of batch-specific tertile distribution of the *IGFBP3 *expression levels separately for each batch and then merged by tertile category. The log-rank test was applied to examine the differences in survival across the comparison groups. Cox proportional hazard models were applied for calculating hazard ratios, using the lowest tertile as a reference group after adjustment for age, TNM (tumor, node, metastasis) stage, chemotherapy, radiotherapy, and tamoxifen use. All *p *values presented are two-sided. SAS software was used for statistical analysis (version 9.1; SAS Institute, Cary, NC, USA).

## Results

The distributions of demographic characteristics and known breast cancer risk factors of 142 patients with breast cancer and 96 patients with BBD are shown in Table 1. The mean age was 48.1 years for patients with breast cancer and 42.7 years for patients with BBD (*p *< 0.001). Patients with breast cancer were more likely to be in their menopause than patients with BBD (*p *< 0.001). Patients with breast cancer showed a higher waist-to-hip ratio (*p *= 0.027) than patients with BBD. The distributions of other breast cancer risk factors were similar in patients with cancer and in those with BBD.

**Figure 1:**
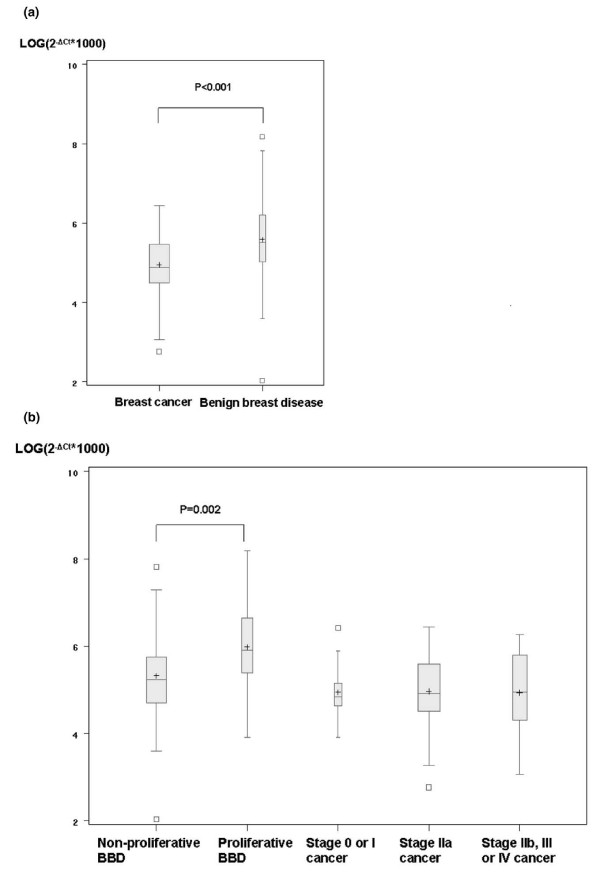
*IGFBP3 *expression in tumor tissues. Results are shown for differences between benign breast disease (BBD) and cancer **(a) **and between grades for BBD and stages for cancer **(b)**. Values of *p *were calculated with a linear regression model including adjustment for age and menopausal status. *Ct*, threshold cycle; *IGFBP3*, gene encoding insulin-like growth factor binding protein 3.

Figure 1 shows the distribution of gene expression in tumor tissues by patient groups. Table 2 presents median and interquartile ranges for the expression levels of the *IGFBP3 *gene in the tumor tissue and adjacent normal tissue of patients with breast cancer or BBD. The expression levels of *IGFBP3 *mRNA were substantially lower in tumor tissues from patients with cancer than those from patients with BBD (*p *< 0.001). In the adjacent normal tissues, however, *IGFBP3 *expression levels were somewhat higher in patients with cancer than in patients with BBD, although the difference was not statistically significant. Among patients with BBD, *IGFBP3 *expression in both tumor and adjacent tissues was higher in patients who had proliferative tumors than those who had non-proliferative tumors. Among patients with cancer, the gene expression levels in both tumor and adjacent normal tissues were elevated in advanced stage cancer; however, the difference was not statistically significant. The expression levels in cancer tissues did not differ by estrogen receptor (ER) or progesterone receptor status. Table 3 presents the study result by menopausal status. No apparent modifying effect by menopausal status was found. The difference in *IGFBP3 *mRNA level by BBD histology groups was not statistically significant in postmenopausal women, perhaps as a result of a small sample size.

*IGFBP3 *expression in tumor tissues was significantly higher than that in their paired adjacent normal tissue among patients with BBD (Table 4; *p *= 0.006). In contrast, in patients with breast cancer, *IGFBP3 *expression levels in the tumor were lower than the adjacent normal tissue, although the difference was not statistically significant.

Table 5 shows the association of *IGFBP3 *expression levels in cancer tissue with breast cancer survival. The median follow-up time for patients with breast cancer was about 7 years. TNM stage was an important prognostic factor. There were no apparent associations of *IGFBP3 *expression with overall survival or disease-free survival. The exclusion of stage IV patients did not change the results. Stratified analyses by menopausal status also yielded similar results for all subjects (data not shown). Additional analyses were performed to include *IGFBP3 *mRNA level (log-transformed) as a continuous variable in the Cox regression model after additional adjustment for the batch effect. Again, no apparent association was found (data not shown).

## Discussion

Most studies on the association between IGFBP-3 and breast cancer risk have been focused on the level of circulating IGFBP-3 [5]. It has been suggested that IGFBP-3 may also have an IGF-independent growth inhibitory effect on breast cancer cells [16,17], and it is important to evaluate tumor tissue specific expressions of *IGFBP3 *level to understand the role of this protein in breast tumorigenesis. Few studies have compared the expression level of the *IGFBP3 *gene in breast cancer tissues with those in adjacent normal tissues or the expression levels across groups of patients with different diagnoses of breast diseases. Pekonen and colleagues [10] reported that mRNA levels of *IGFBP3 *and other IGFBPs were significantly higher in five breast cancer tissues than in adjacent normal tissues as measured by Western blotting. However, Nardon and colleagues [9] did not find a significant difference in IGFBP-3 expression between breast tissues from 15 patients with diabetes without breast cancer and breast tumor tissues from 19 patients with breast cancer by using immunohistochemistry. We found that *IGFBP3 *mRNA levels were substantially lower in cancer tissues than in tumor tissues from patients with BBD. In patients with BBD, *IGFBP3 *mRNA levels were elevated in tumor tissues, particularly from patients with a proliferative BBD, compared with adjacent normal tissues.

Although no other study has systematically evaluated *IGFBP3 *expression as in our study, results from a study of prostate tumor provide some support to our finding. Tennant and colleagues [18] compared the expression of IGFBP-3 in prostate tissue containing benign epithelium, high-grade prostate intraepithelial neoplasia (PIN), and adenocarcinoma. They reported a significantly increased IGFBP-3 immunoreactivity in PIN regions compared with normal epithelium, and a significant decrease in malignant cells. However, *IGFBP3 *mRNA levels remained virtually unchanged in benign epithelium, PIN, and adenocarcinoma cells, indicating a pre-translational and/or post-translational modification of IGFBP-3 [18]. Breast cancer cells secrete various types of IGFBP, and the expression of IGFBP-3 is hormonally regulated [16]. The reduced expression of *IGFBP3 *in cancer tissues is consistent with the role of IGFBP-3 in cancer inhibitory effects. The elevated expression of *IGFBP3 *in proliferative benign breast tumor is unexpected. However, it has been suggested that both transcriptional and post-transcriptional regulation are involved for the regulation of IGFBP-3 expression [1,19]. Moreover, various growth factors are also involved in the expression of IGFBP-3 [16,20,21]. The role of IGFBP-3 expression in breast tumors should therefore be understood in the context of complex regulation for cancer cell growth.

The level of *IGFBP3 *mRNA was found to be higher in ER-negative than in ER-positive tumors in one [22] of the two studies [10,22] conducted previously. We failed to find this association in our study with a larger sample size. In contrast, studies measuring IGFBP-3 protein levels in a series of human breast cancer tissues showed higher IGFBP-3 levels in ER-negative than in ER-positive tumors [11,23-26]. It seems that the association observed might depend on the method used to detect IGFBP-3 level in breast tissue [2].

Several [10,11,22-25,27,28] but not all [10,26] previous studies have shown that a high tissue level of IGFBP-3 is related to unfavorable prognostic factors of breast cancer, such as large tumor size, elevated S-phase fraction, elevated DNA aneuploidy, and low levels of ER or progesterone receptor. High expression of *IGFBP3 *was directly associated with poor overall survival in one study [26] but not in another [23], and it was not associated with the recurrence of breast cancer [23,26]. We did not find that *IGFBP3 *expression was associated with breast cancer survival in our study, which is consistent with one of only two previous studies. However, most studies included only a small number of subjects, ranging between 8 and 200 tumor samples from patients with breast cancer. Moreover, none of the studies have directly evaluated the association between *IGFBP3 *mRNA expression and breast cancer survival. Our finding therefore needs to be confirmed in other large studies.

IGFBP-3 acts as a direct growth inhibitor and induces apoptosis by an IGF-independent mechanism. It has been shown that IGFBP-3 inhibits the growth of the IGF-unresponsive Hs578 human breast cancer cell line [8,16] and enhances the effects of ceramide and paclitaxol-induced apoptosis directly [7,29]. In the prostate cancer cell line PC-3, the addition of exogenous IGFBP-3 resulted in a dose-dependent increase in the apoptotic index, which was only partly attenuated by the addition of IGF-I [30]. By contrast, IGFBP-3 was also shown to have a potential mitogenic effect by enhancing epidermal growth factor signaling, phosphorylation of the epidermal growth factor receptor, and the Ras-p44/42 and p38 mitogen-activated protein kinase (MAPK) signaling pathways [20,31]. The decreased *IGFBP3 *expression observed in our study of cancer tissues is consistent with these cancer-inhibitory effects of IGFBP-3.

We also evaluated gene expression patterns of other IGF system components, including IGF-I and IGF-1 receptor (IGF1R) in our study [32]. Patients with BBD had a significantly higher *IGF-I *gene expression than patients with breast cancer in both tumor and adjacent non-neoplastic tissues. Interestingly, the expression levels of *IGF-I *and *IGFIR *in cancer patients were substantially higher in tumor-adjacent tissues than in tumor tissues. Patients with a high expression of *IGF-I *in cancer tissues showed more favorable overall and disease-free survival [32].

In summary, we found that cancer tissues from patients with breast cancer had significantly lower *IGFBP3 *expression than tumor tissues from patients with BBD, and *IGFBP3 *expression in both tumor and adjacent normal tissues were higher in tissues from patients with proliferative BBD than in those from patients with non-proliferative BBD. Our findings increase our understanding of the mechanisms of IGFBP-3 in the pathogenesis of breast cancer and suggest the need for further molecular genetic studies to elucidate the biological significance of the abnormal expression of *IGFBP3 *in breast lesions.

## Conclusion

*IGFBP3 *gene expression was systematically evaluated in tumor tissue and adjacent normal tissues from patients diagnosed with breast cancer or BBD. The expression level of *IGFBP3 *was higher in tumor tissues from BBDs than in those from cancer. However, *IGFBP3 *expression was not associated with breast cancer survival.

## Abbreviations

BBD = benign breast disease; *Ct *= threshold cycle; ER = estrogen receptor; IGF = insulin-like growth factor; IGF1R = IGF-1 receptor; IGFBP-3 = insulin-like growth factor binding protein 3; PCR = polymerase chain reaction; PIN = prostate intraepithelial neoplasia; TNM = tumor, node, metastasis.

## Competing interests

The authors declare that they have no competing interests.

## Authors' contributions

ZR performed the laboratory assays. AS performed the statistical analyses. ZR, AS, and WZ drafted the manuscript. QC, XOS, and YTG critically reviewed the manuscript and contributed to its revision. XO, YTG, and WZ contributed to the study design and data/sample collection. WZ was the principal investigator of the study. All authors read and approved the final manuscript.

## Figures and Tables

**Table 1:** Demographic and selected breast cancer risk factors for patients with breast cancer and with BBD

Characteristics^a^	Breast cancer (*n *= 142)	BBD (*n *= 96)	*p*
Demographic factors			
Age (years)	48.1 ± 7.4	42.7 ± 6.7	<0.001
High school education or higher (%)	45.1	49.0	0.555
Known risk factors			
Age at menarche	14.6 ± 1.7	14.7 ± 1.7	0.740
Postmenopause (%)	35.5	7.4	<0.001
Age at menopause^b^	48.7 ± 4.3	44.6 ± 7.9	0.222
Breast cancer history among first-degree relatives (%)	4.2	3.1	0.662
Ever used hormone replacement therapy (%)	3.5	1.0	0.231
Body mass index (kg/m^2^)	23.9 ± 3.0	23.3 ± 3.4	0.193
Waist-to-hip ratio	0.81 ± 0.05	0.79 ± 0.05	0.027
Ever consumed alcohol (%)	2.8	2.1	0.723
Physically active during past 10 years (%)	19.0	12.5	0.183

BBD, benign breast disease. ^a ^Values are presented in means ± Standard Deviation (unless otherwise noted). ^b^Among postmenopausal women.

**Table 2:** *IGFBP3 *mRNA levels for patients with breast cancer and with BBD

Parameter	Tumor tissue		Adjacent tissue	
	No. of patients	*IGFBP3 *mRNA level	No. of patients	*IGFBP3 *mRNA level
BBD	96	248.1 (152.2, 496.5)	60	178.9 (104.0, 322.7)
Breast cancer	142	132.3 (89.3, 235.2)	63	205.6 (157.1, 301.5)
*p*^a^		<0.001		0.763
BBD				
Non-proliferative lesions	57	185.4 (109.6, 314.2)	34	157.4 (100.2, 243.2)
Proliferative lesions	39	367.0 (217.2, 771.3)	26	218.1 (137.6, 394.3)
*p*^a^		0.002		0.044
Breast cancer (by stage)				
0 and I	32	125.9 (103.0, 173.2)	21	214.9 (106.4, 262.6)
IIa	63	135.7 (89.6, 268.5)	27	205.6 (158.9, 362.9)
IIb, III and IV	47	141.1 (73.7, 328.9)	15	199.4 (164.4, 320.7)
*p *for trend test^a^		0.924		0.448
Breast cancer (by ER status)				
Positive	75	133.0 (89.3, 230.6)	33	208.1 (165.1, 301.5)
Negative	36	136.0 (77.6, 237.3)	19	192.1 (155.2, 277.1)
Unknown	31	125.8 (90.3, 359.0)	11	214.9 (145.4, 742.1)
*p*^a^		0.873		0.376
Breast cancer (by PR status)				
Positive	74	131.3 (89.3, 230.6)	34	208.2 (167.1, 301.5)
Negative	36	136.3 (77.6, 209.4)	17	169.4 (155.2, 274.6)
Unknown	32	136.0 (90.8, 355.4)	12	193.5 (125.9, 534.7)
*p*^a^		0.710		0.472

Results for *IGFBP3 *mRNA levels are shown as median (25th, 75th centile). BBD, benign breast disease; ER, estrogen receptor, *IGFBP3*, gene encoding insulin-like growth factor binding protein 3; PR, progesterone receptor. ^a^Adjusted for age and menopausal status.

**Table 3:** *IGFBP3 *mRNA levels for patients with breast cancer and with BBD, stratified by menopausal status

Status	Disease	Tumor tissue		Adjacent tissue	
		No. of patients	*IGFBP3 *mRNA level	No. of patients	*IGFBP3 *mRNA level
Premenopausal	BBD	88	231.6 (147.8, 488.0)	54	178.9 (104.6, 300.3)
	Breast cancer	91	146.2 (85.2, 289.2)	41	199.4 (156.6, 274.1)
	*p*^a^		<0.001		0.472
	BBD				
	Non-proliferative lesions	53	185.4 (109.6, 279.4)	31	139.2 (100.2, 243.2)
	Proliferative lesions	35	353.1 (197.9, 709.3)	23	219.1 (141.8, 394.3)
	*p*^a^		0.006		0.055
	Breast cancer (by stage)				
	0 and I	23	125.0 (102.7, 169.0)	16	203.1 (85.4, 263.9)
	IIa	36	196.5 (86.7, 337.6)	16	200.9 (126.8, 344.1)
	IIb, III & IV	32	139.1 (78.9, 301.8)	9	199.4 (165.1, 235.0)
	*p *for trend test^a^		0.775		0.740
Postmenopausal	BBD	7	314.2 (183.9, 528.5)	5	201.8 (73.9, 390.0)
	Breast cancer	50	121.8 (96.3, 223.2)	21	208.2 (167.1, 362.8)
	*p*^a^		0.006		0.184
	BBD				
	Non-proliferative lesions	4	249.1 (119.4, 421.3)	3	201.8 (29.5, 390.0)
	Proliferative lesions	3	472.6 (274.5, 1500.8)	2	239.5 (73.9, 405.1)
	*p*^a^		0.157		0.763
	Breast cancer (by stage)				
	0 and I	9	126.7 (116.8, 220.8)	5	221.0 (155.2, 262.6)
	IIa	26	112.1 (96.3, 202.2)	10	206.9 (179.0, 382.1)
	IIb, III & IV	15	141.1 (72.7, 351.8)	6	243.9 (159.0, 727.6)
	*p *for trend test^a^		0.670		0.308

Results for *IGFBP3 *mRNA levels are shown as median (25th, 75th centile). BBD, benign breast disease; *IGFBP3*, gene encoding insulin-like growth factor binding protein 3. ^a^Adjusted for age.

**Table 4:** Comparison of *IGFBP3 *mRNA between tumor tissues and tumor-adjacent tissues

Patient group	Number of pairs	*IGFBP3 *mRNA expression level		*p*^a^
			
		Tumor tissue	Adjacent tissue	
Benign breast disease	60	240.7 (152.1, 533.5)	178.9 (104.0, 322.7)	0.006
Breast cancer	63	165.1 (103.4, 289.2)	205.6 (157.1, 301.5)	0.246

Results for *IGFBP3 *mRNA levels are shown as median (25th, 75th centile). *IGFBP3*, gene encoding insulin-like growth factor binding protein 3. ^a^*p *from paired *t *test.

**Table 5:** Association between *IGFBP3 *mRNA expression and breast cancer survival

Characteristic	*n*	Overall survival		Disease-free survival	
		No. of events	HR (95% CI)	No. of events	HR (95% CI)
Age at diagnosis					
28–39 years	68	16	1.0 (reference)	21	1.0 (reference)
40–49 years	264	48	0.8 (0.4–1.3)	59	0.7 (0.4–1.1)
50–59 years	136	37	1.2 (0.6–2.1)	44	1.0 (0.6–1.7)
60–64 years	53	10	0.7 (0.3–1.6)	12	0.7 (0.3–1.4)
TNM stage					
0, I	124	8	1.0 (reference)	13	1.0 (reference)
IIa	208	39	2.6 (1.3–5.2)	51	2.4 (1.3–4.2)
IIb	132	35	3.9 (1.9–7.8)	39	2.8 (1.6–5.1)
III, IV	49	27	9.8 (4.7–20.3)	31	8.5 (4.6–15.7)
*IGFBP3 *(all subjects)^a^					
T1	173	37	1.0 (reference)	44	1.0 (reference)
T2	174	43	1.2 (0.7–1.8)	55	1.3 (0.9–2.0)
T3	174	31	0.9 (0.5–1.4)	37	0.9 (0.6–1.4)
*p *for trend			0.596		0.715
*IGFBP3 *(excluding stage IV cases)^a^					
T1	172	37	1.0 (reference)	44	1.0 (reference)
T2	171	42	1.1 (0.7–1.9)	53	1.2 (0.8–1.9)
T3	170	30	0.9 (0.5–1.4)	37	0.9 (0.6–1.4)
*p *for trend			0.591		0.777

CI, confidence interval; HR, hazard ratio; *IGFBP3*, gene encoding insulin-like growth factor binding protein 3; TNM, tumor, node, metastasis. ^a^Adjusted for age, stages, chemotherapy, radiotherapy, and tamoxifen use.
